# Melatonin enhances the developmental competence of porcine somatic cell nuclear transfer embryos by preventing DNA damage induced by oxidative stress

**DOI:** 10.1038/s41598-017-11161-9

**Published:** 2017-09-11

**Authors:** Shuang Liang, Yong-Xun Jin, Bao Yuan, Jia-Bao Zhang, Nam-Hyung Kim

**Affiliations:** 1 Department of Laboratory Animals, College of Animal Sciences, Jilin University, Changchun, 130062 Jilin China; 2 Department of Animal Science, Chungbuk National University, Cheongju, Chungbuk 361-763 Republic of Korea

## Abstract

Melatonin has antioxidant and scavenger effects in the cellular antioxidant system. This research investigated the protective effects and underlying mechanisms of melatonin action in porcine somatic cell nuclear transfer (SCNT) embryos. The results suggested that the developmental competence of porcine SCNT embryos was considerably enhanced after melatonin treatment. In addition, melatonin attenuated the increase in reactive oxygen species levels induced by oxidative stress, the decrease in glutathione levels, and the mitochondrial dysfunction. Importantly, melatonin inhibited phospho-histone H2A.X (γH2A.X) expression and comet tail formation, suggesting that γH2A.X prevents oxidative stress-induced DNA damage. The expression of genes involved in homologous recombination and non-homologous end-joining pathways for the repair of double-stranded breaks (DSB) was reduced upon melatonin treatment in porcine SCNT embryos at day 5 of development under oxidative stress condition. These results indicated that melatonin promoted porcine SCNT embryo development by preventing oxidative stress-induced DNA damage via quenching of free radical formation. Our results revealed a previously unrecognized regulatory effect of melatonin in response to oxidative stress and DNA damage. This evidence provides a novel mechanism for the improvement in SCNT embryo development associated with exposure to melatonin.

## Introduction

Somatic cell nuclear transfer (SCNT) is an extraordinary and important technology for generating transgenic animals and preserving species^1–3^. Although several mammalian species have been successfully cloned using SCNT technology^4–6^, its success rate remains extremely low, especially in pigs^7^. One of the underlying problems during the production of SCNT embryos is the incorrect or incomplete epigenetic reprogramming^8, 9^. To overcome these problems, many chemicals associated with epigenetic modifications have been used. For instance, DNA methyltransferase inhibitors such as BIX-01294 and 5′-azacytidine (5′-azaC), and histone deacetylase inhibitors such as trichostatin A, scriptaid, and valproic acid have greatly improved the cloning efficiency in several species^9–13^. Another major problem during the production of SCNT embryos is the variation in culture conditions. Although many advances have been made in embryo cell culture techniques, the proportion of embryos, especially SCNT embryos, that develop to the blastocyst stage is still variable^14–16^.

The physiological levels of intracellular reactive oxygen species (ROS) play a key role in maintaining embryonic development^17, 18^. However, an excessive accumulation of ROS is correlated with defective embryo development^19–21^. Many studies suggested that a significant increase in ROS production during embryo development *in vitro* contributed to embryonic DNA damage and mitochondrial dysfunction^21–23^. After SCNT, the donor cell’s recipient undergoes chromatin remodeling and epigenetic reprogramming^8^. Therefore, to efficiently maintain genomic integrity and ensure genomic stability, cell reprogramming must occur properly for SCNT embryos to develop^24^. Previous studies showed that SCNT embryos exhibited more DNA damage than embryos generated by *in vitro* fertilization (IVF)^25, 26^. In addition, DNA damage inhibits the reprogramming of differentiated cells into induced pluripotent stem (iPS) cells^27^. In somatic cells, the increasing histone acetylation by histone deacetylase inhibitor (HDACi) treatment has a beneficial role in DNA damage repair^28, 29^. The HDACi scriptaid effectively enhances DNA damage repair and improves somatic cell reprogramming by increasing the ability of SCNT embryos to develop *in vitro* ^30^. Thus, inhibition of DNA damage is an effective strategy for improving the developmental competence of SCNT embryos.

Melatonin (N-acetyl-5-methoxytryptamine), a hormone mainly synthesized in the pineal gland, has multiple effects on different physiological processes^31^. Melatonin plays a key role in a variety of important physiological functions by acting as an antioxidant and free radical scavenger^32, 33^. Previous studies have indicated that melatonin not only increases the activities of antioxidative enzymes but also inhibits pro-oxidative enzymes by reducing cellular oxidative damage^34, 35^. Melatonin is effective in promoting the formation of pluripotent embryonic stem (ES) cell lines in buffaloes^36^. In addition, the beneficial effects of melatonin supplementation in mammalian embryos produced *in vitro* have been described^15, 37–39^. A recent study demonstrated that melatonin improves the developmental competence of porcine embryos by inhibiting the p53-mediated apoptotic pathway^37^. Moreover, Su et al^15^. demonstrated that supplementation with exogenous melatonin reduced apoptosis and levels of ROS in bovine SCNT embryos, which improved overall cloning efficiency.

Although melatonin enhances chromatin reprogramming and increases the efficiency of SCNT embryo generation^15, 37, 40^, its mechanisms of action are not fully understood. In the present study, we hypothesized that melatonin enhanced the developmental competence of porcine SCNT embryos by preventing oxidative stress-induced DNA damage. Our research expands the understanding of the role of melatonin in SCNT embryo development.

## Results

### Effects of treatment with various concentrations of melatonin on *in vitro* development of porcine SCNT embryos

**Figure 1:**
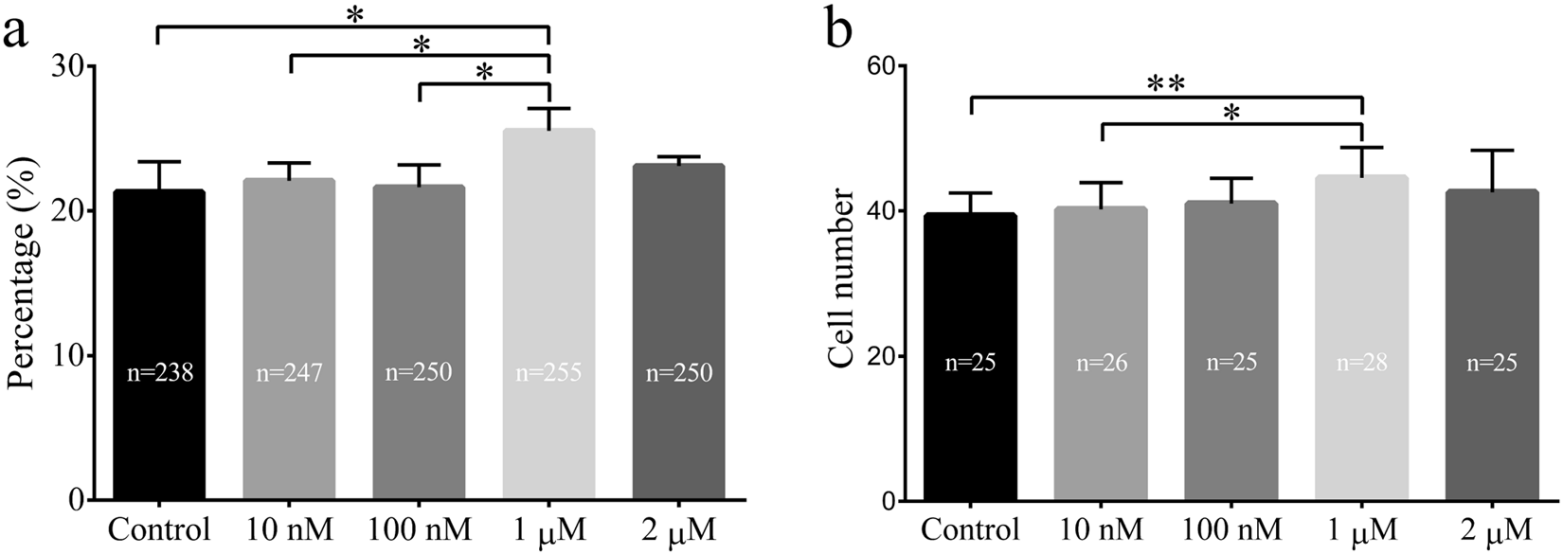
Effects of melatonin on the *in vitro* development of porcine somatic cell nuclear transfer (SCNT) embryos. Blastocyst formation rates (**a**) and overall cell numbers (**b**) derived from porcine SCNT embryos cultured in the presence of melatonin at various concentrations. The numbers of embryos examined in each group are shown in the bars. Data are expressed as the mean ± standard deviation (SD) from at least three separate experiments. *p < 0.05; **p < 0.01.

**Figure 2:**
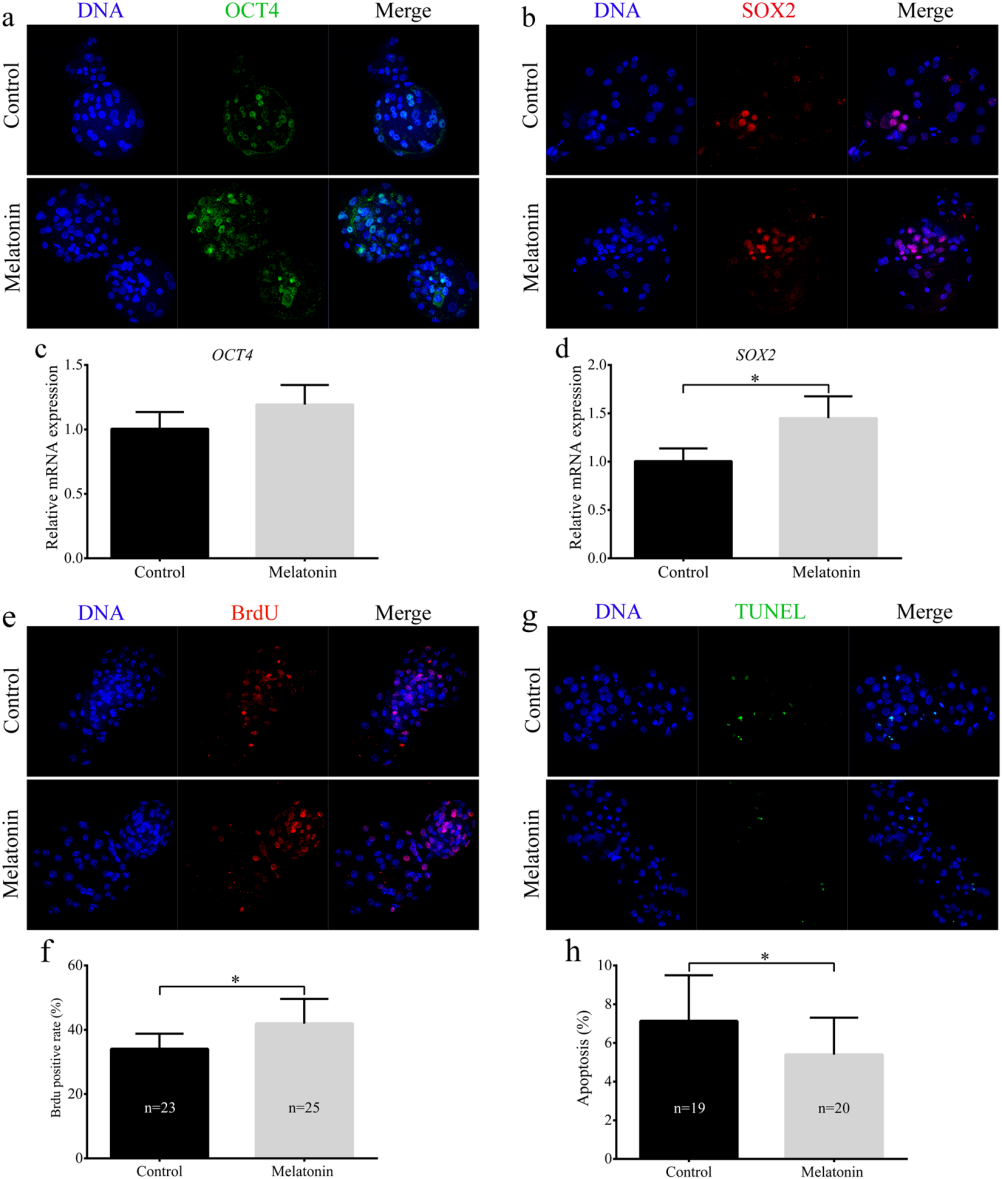
Effects of melatonin treatment on expression of pluripotency genes, cell proliferation, and apoptosis in porcine somatic cell nuclear transfer (SCNT) embryos. Immunofluorescent staining of octamer-binding transcription factor 4 (OCT4) (**a**) and SOX2 (**b**) in blastocysts. Relative expression levels for *OCT4* (**c**) and *SOX2* (**d**) in blastocysts developing *in vitro* in the presence or absence melatonin. (**e**) Immunofluorescent staining of 5-bromo-2′-deoxyuridine (BrdU) in blastocysts. (**f**) Rates of cell proliferation in blastocysts developing *in vitro* in the presence or absence melatonin. (**g**) Terminal deoxynucleotidyl transferase (TdT) 2′-deoxyuridine, 5′-triphosphate (dUTP) nick end labeling (TUNEL)-positive cells were detected in blastocysts. (**h**) Proportion of apoptotic cells in blastocysts developing *in vitro* in the presence or absence melatonin. The numbers of embryos examined in each group are shown in the bars. Data are expressed as the mean ± standard deviation (SD) from at least three separate experiments. *p < 0.05.

We assayed whether melatonin improved the developmental competence of porcine SCNT embryos. After activation, SCNT embryos were treated with different concentrations of melatonin (10 nM, 100 nM, 1 μM, or 2 μM), and their embryonic development was examined *in vitro*. The results showed that treatment with 1 μM melatonin significantly increased the rate of blastocyst formation for the SCNT embryos (25.5% ± 1.6% vs. 21.3% ± 2.1%, *p* < 0.05) (Fig. 1a) and increased the total cell numbers (44.5% ± 4.2% vs. 39.3% ± 3.1%, *p* < 0.05) compared to those cultured in the untreated group (Fig. 1b). Further analyses revealed that 1 μM melatonin treatment not only increased expression of pluripotency markers (SOX2) and cell proliferation but also decreased apoptosis in these embryos (*p* < 0.05, Fig. 2). Based on these results, 1 μM melatonin was used in all subsequent experiments.

### Melatonin protected the development of porcine SCNT embryos against oxidative stress induced by H_2_O_2_

Since melatonin is a free radical scavenger and has antioxidant properties^32^, we hypothesized that melatonin increased the resistance of embryos to oxidative stress. To induce oxidative stress, H_2_O_2_ was used to treat porcine SCNT embryos after activation. When the SCNT embryos were exposed to 100 μM H_2_O_2_, the rates of blastocyst formation decreased significantly (18.7% ± 0.5% vs. 21.5% ± 1.1%, *p* < 0.05). Hence, the role of melatonin during the development of porcine SCNT embryos was investigated after treatment with 100 μM H_2_O_2_. Melatonin treatment rescued the developmental potential of embryos after H_2_O_2_-induced stress. When the embryos were treated with melatonin, the proportion of embryos reaching the blastocyst stage was rescued (21.3% ± 0.7% vs. 18.7% ± 0.5%, *p* < 0.05, Supplemental Figure S1).

### Melatonin decreased intracellular ROS level, increased intracellular glutathione (GSH) level, and prevented mitochondrial dysfunction induced by H_2_O_2_ in porcine SCNT embryo**s**

**Figure 3:**
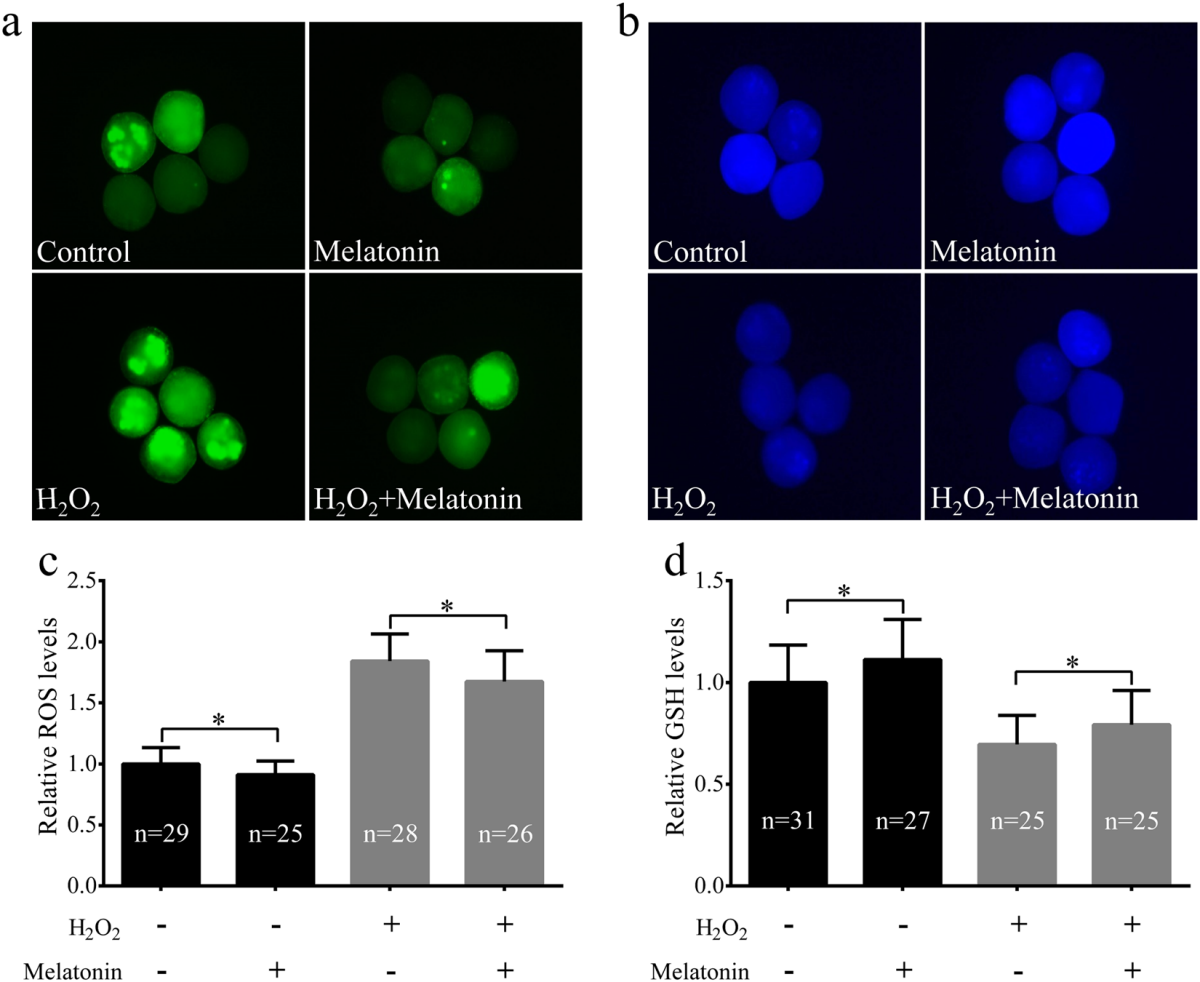
Melatonin reduces H_2_O_2_-induced intracellular reactive oxygen species (ROS) levels and increases glutathione (GSH) levels in porcine somatic cell nuclear transfer (SCNT) embryos. Representative fluorescent images of ROS (**a**) and GSH (**b**) in porcine SCNT embryos at the one-cell stage in the presence or absence of melatonin under H_2_O_2_-induced oxidative stress. Quantification of ROS (**c**) and GSH (**d**) levels in porcine SCNT embryos. The numbers of embryos examined in each group are shown in the bars. Data are expressed as the mean ± standard deviation (SD) from at least three separate experiments. *p < 0.05.

**Figure 4:**
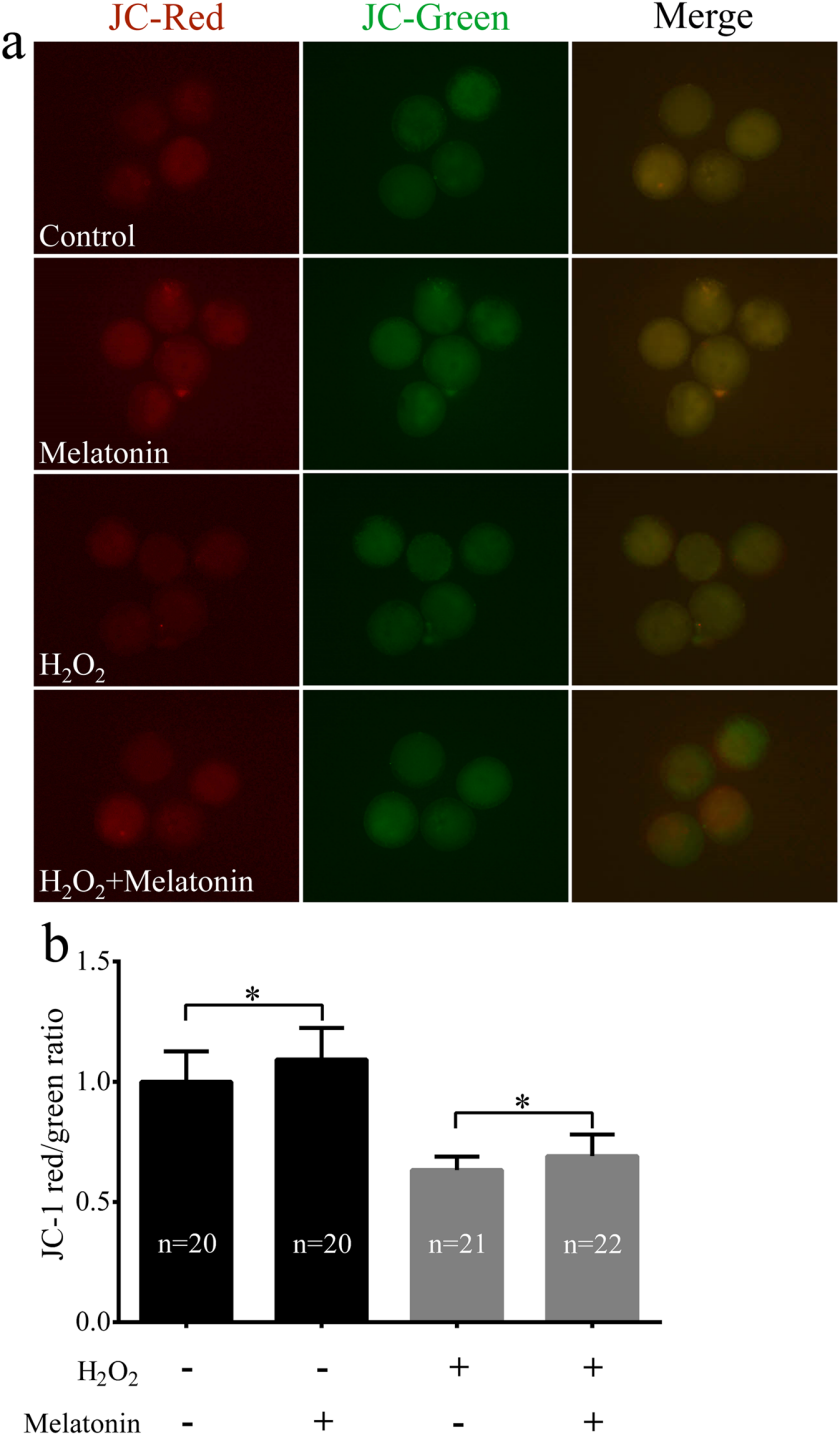
Melatonin treatment prevented H_2_O_2_-induced mitochondrial dysfunction in porcine somatic cell nuclear transfer (SCNT) embryos. (**a**) Representative fluorescent images of 5,5′,6,6′-tetrachloro-1,1′,3,3′-tetraethyl-imidacarbocyanine iodide (JC-1) staining in porcine SCNT embryos at the one-cell stage in the presence or absence of melatonin under H_2_O_2_-induced oxidative stress. (**b**) Quantification of relative fluorescence intensity of JC-1 (**c**) in porcine SCNT embryos. The numbers of embryos examined in each group are shown in the bars. Data are expressed as the mean ± standard deviation (SD) from at least three separate experiments. *p < 0.05.

To determine whether melatonin rescued the damage associated with oxidative stress in porcine SCNT embryos, ROS and GSH levels at the one-cell stage were quantified. As shown in Fig. 3a and c, embryos co-treated with melatonin displayed significantly lower ROS levels than the H_2_O_2_-treated embryos (*p* < 0.05). In addition, compared to the H_2_O_2_-treated embryos, embryos co-treated with melatonin produced higher levels of GSH (*p* < 0.05, Fig. 3b and d). Since the mitochondria plays a key role in the production of ROS, its dysfunction compromises embryo development^41^. Thus, the mitochondrial membrane potential (ΔΨm) was also evaluated in porcine SCNT embryos after H_2_O_2_ treatment. Representative images of mitochondrial ΔΨm are shown in Fig. 4a. After exposing porcine SCNT embryos to H_2_O_2_, the levels of ΔΨm were rapidly reduced, whereas treatment with melatonin restored ΔΨm in the embryos (*p* < 0.05, Fig. 4b). In addition, in the non-treated H_2_O_2_ group, melatonin effectively increased the levels of ΔΨm in porcine SCNT embryos (*p* < 0.05).

### Melatonin reduced phospho-histone H2A.X (γH2A.X) and led to increased global histone acetylation induced by H_2_O_2_ in porcine SCNT embryos

**Figure 5:**
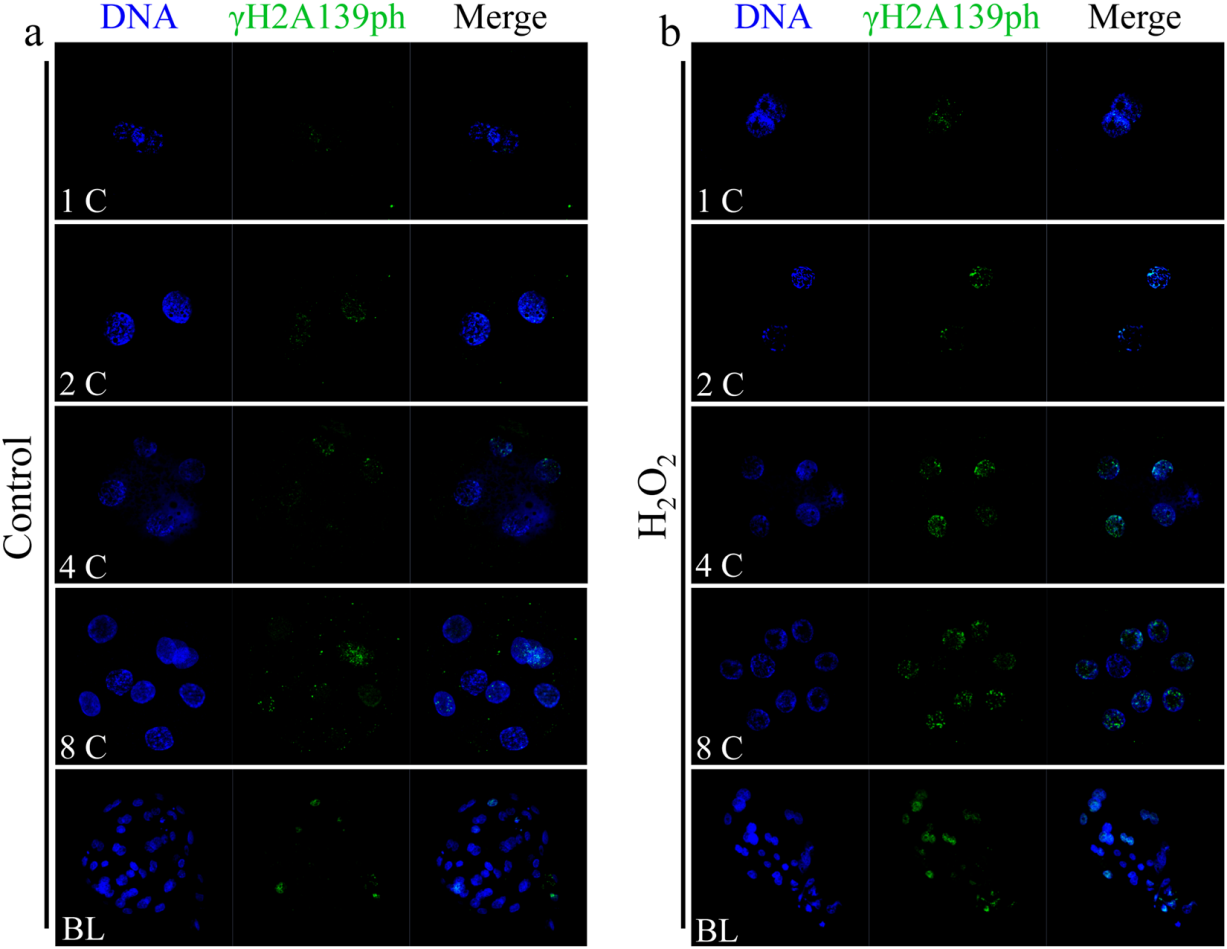
Treatment with H_2_O_2_ increases the presence of phospho-histone H2A.X (γH2A.X) at sites of DNA damage. Representative fluorescent images show the presence of γH2A.X in the nuclei of control somatic cell nuclear transfer (SCNT) embryos (**a**) or SCNT embryos (**b**) exposed to H_2_O_2_ at different developmental stages.

**Figure 6:**
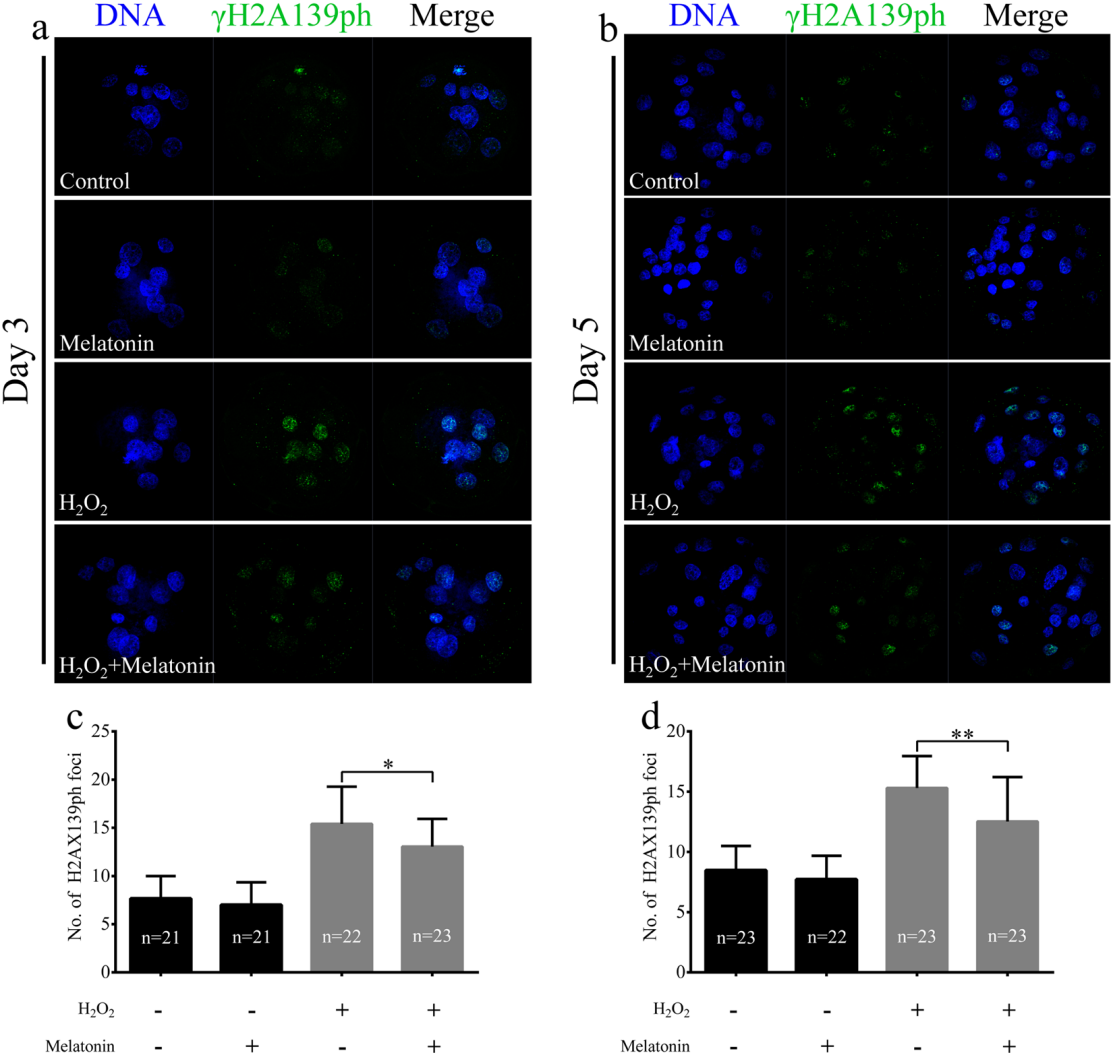
Melatonin attenuates H_2_O_2_-induced DNA damage in porcine somatic cell nuclear transfer (SCNT) embryos. Representative fluorescent images of phospho-histone H2A.X (γH2A.X) in the nuclei of porcine SCNT embryos at 3 (**a**) and 5 days (**b**) of culture in the presence or absence of melatonin under H_2_O_2_-induced oxidative stress. The average number of fluorescent γH2A.X foci in the nuclei of porcine SCNT embryos at 3 (**c**) and 5 days (**d**) of culture. The numbers of embryos examined in each group are shown in the bars. Data are expressed as the mean ± standard deviation (SD) from at least three separate experiments. *p < 0.05; **p < 0.01.

**Figure 7:**
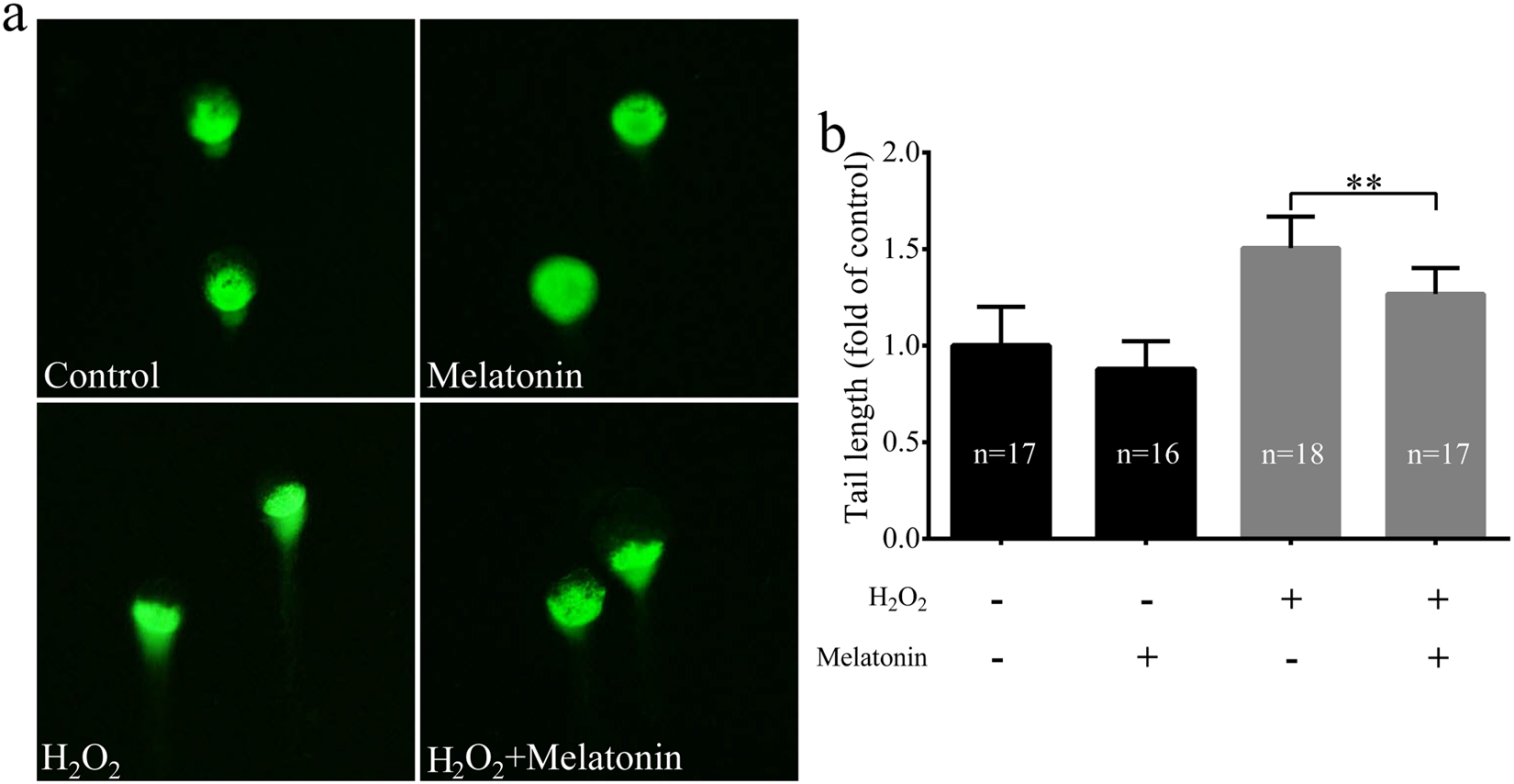
Melatonin attenuates H_2_O_2_-induced DNA damage in porcine somatic cell nuclear transfer (SCNT) embryos as revealed by the comet assay. (**a**) Representative fluorescent images of comet assay in porcine SCNT embryos at 3 days of culture. (**b**) Fold changes in tail moment length of porcine SCNT embryos at 3 days of culture. The comet head is made up of undamaged DNA, while the comet tail is composed of damaged DNA. Embryo comets are visualized with a fluorescence microscope and analyzed to calculate the length of the tail. The numbers of embryos examined in each group are shown in the bars. Data are expressed as the mean ± standard deviation (SD) from at least three separate experiments. **p < 0.01.

**Figure 8:**
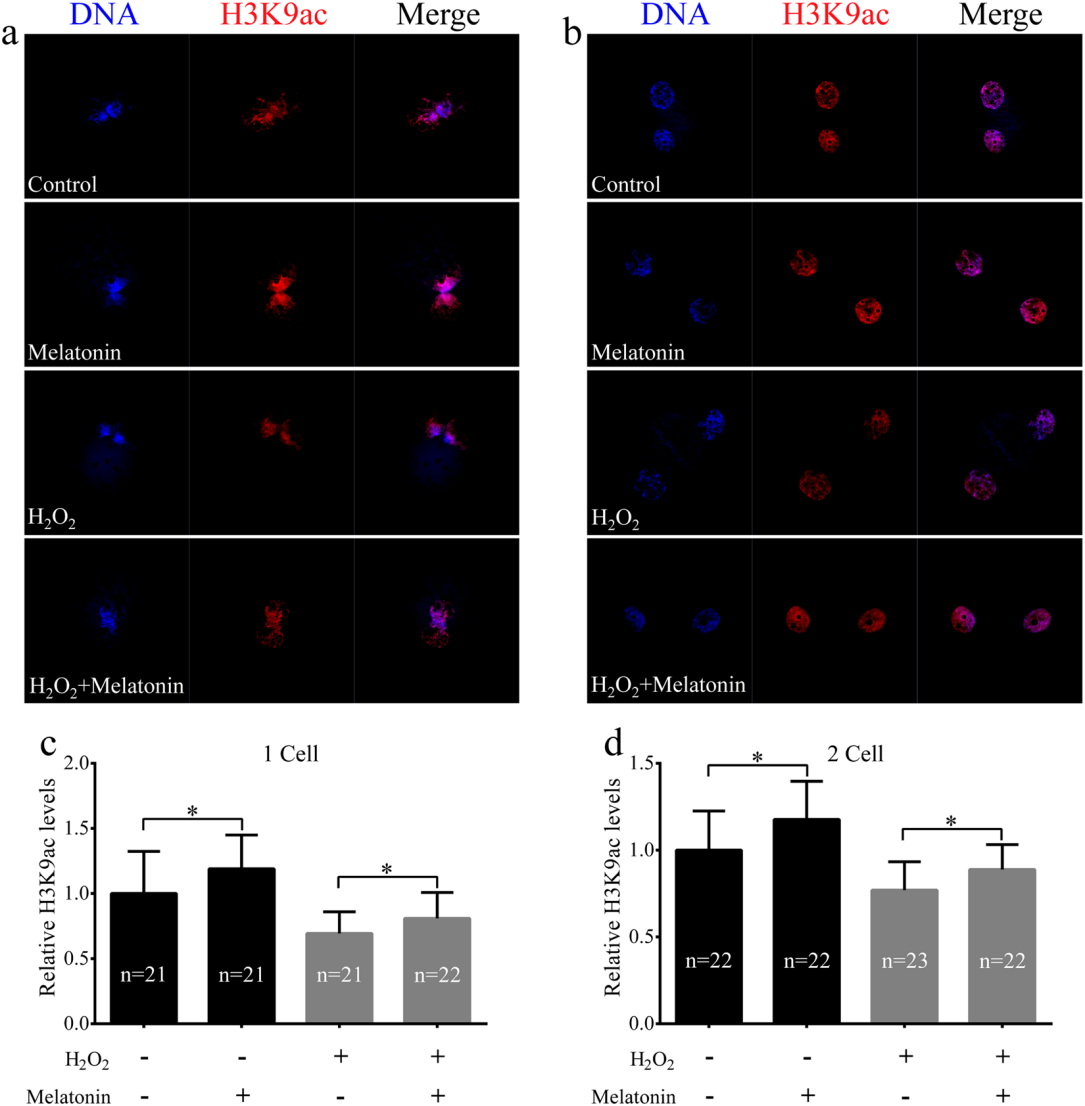
Melatonin treatment increases global histone 3 lysine 9 acetylation (H3K9ac) levels in porcine somatic cell nuclear transfer (SCNT) embryos. Representative fluorescent images of H3K9ac in porcine SCNT embryos at the one-cell (**a**) and two-cell (**b**) stages in the presence or absence of melatonin under H_2_O_2_-induced oxidative stress. Quantification of H3K9ac levels in porcine SCNT embryos at the one-cell (**c**) and two-cell (**d**) stages. The numbers of embryos examined in each group are shown in the bars. Data are expressed as the mean ± standard deviation (SD) from at least three separate experiments. *p < 0.05.

**Figure 9:**
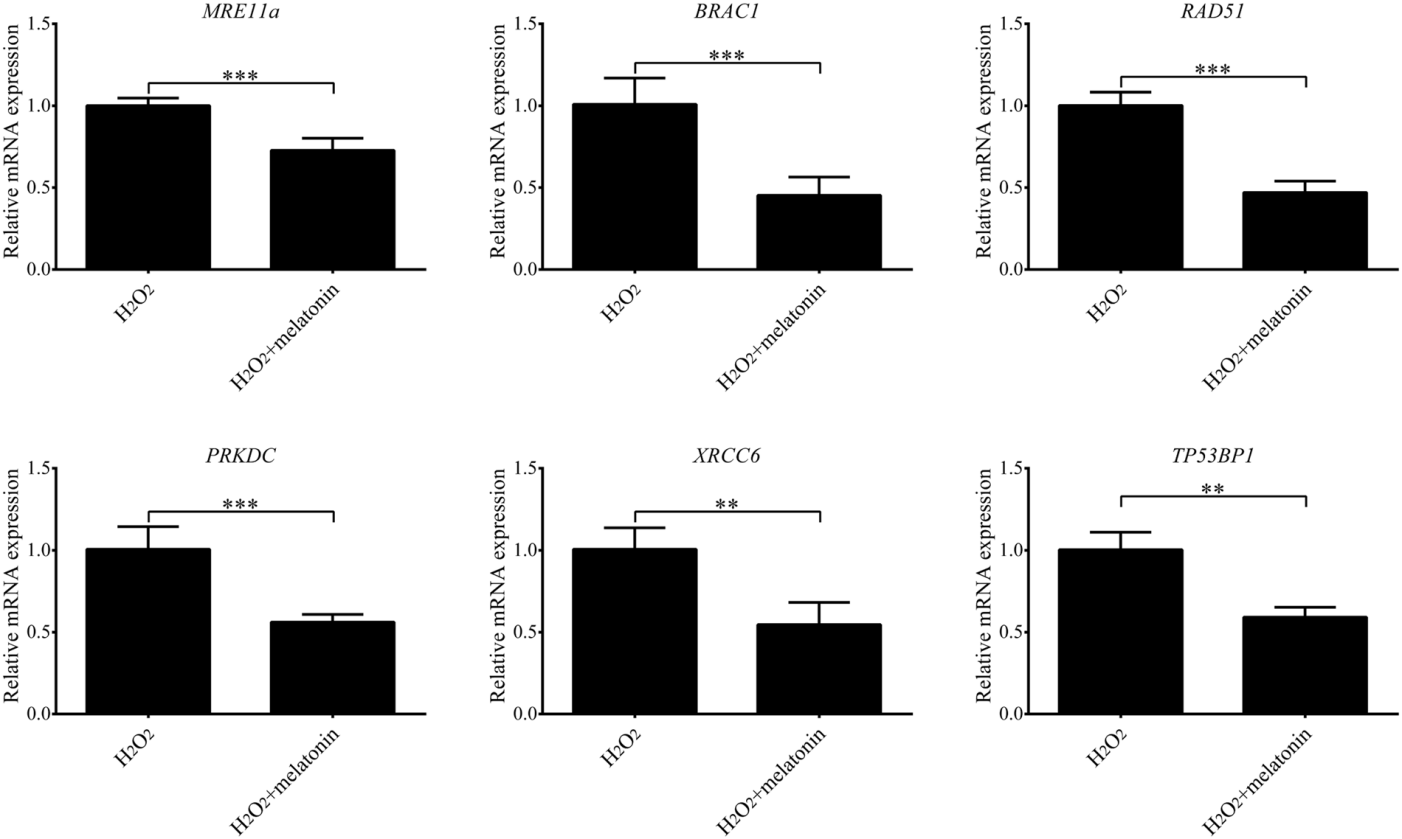
Relative mRNA levels of damage-related genes of the homologous recombination (HR) and non-homologous end-joining (NHEJ) pathways in somatic cell nuclear transfer (SCNT) embryos at 5 days of development. Data are expressed as the mean ± standard deviation (SD) from at least three separate experiments. **p < 0.01; ***p < 0.001.

A previous study demonstrated that SCNT embryos exhibited higher levels of DNA damage compared to IVF embryos^42^. We detected the expression of γH2A.X, a marker of DNA repair after DNA damage, in porcine SCNT embryos treated with H_2_O_2_. H_2_O_2_-treated SCNT embryos expressed higher levels of γH2A.X than those of non-treated SCNT embryos (Fig. 5). We determined whether melatonin exerted protective effects against DNA damage after H_2_O_2_ exposure in porcine SCNT embryos. We observed that there was a clear decrease in the expression of γH2A.X in porcine SCNT embryos after treatment with H_2_O_2_ in the presence of melatonin at 3 and 5 days of development (*p* < 0.05, Fig. 6). The DNA comet assay confirmed that the repair of H_2_O_2_-induced DNA damage increased in the presence of melatonin (*p* < 0.05, Fig. 7). Furthermore, we quantified the levels of global histone acetylation in porcine SCNT embryos at the one- and two-cell stages (Fig. 8a and b). Our results showed that the global histone 3 lysine 9 acetylation (H3K9ac) levels were significantly higher in melatonin-treated SCNT embryos than those in non-treated embryos (*p* < 0.05, Fig. 8c and d). To further explore the effect of melatonin treatment on DNA damage repair, we examined the expression of genes involved in homologous recombination (HR) and non-homologous end-joining (NHEJ) in SCNT embryos at 5 and 7 days of development. At 5 days of development in SCNT embryos after H_2_O_2_ exposure, melatonin treatment reduced the mRNA expression levels of both HR-related genes (*MRE11a*, *BRAC1*, and *RAD51*) and NHEJ-related genes (*PRKDC*, *XRCC6*, and *TP53BP1*), which were involved in the repair of DNA damage (*p* < 0.05, Fig. 9). However, there was no difference in the mRNA expression levels of genes involved in the HR and NHEJ repair pathways at 7 days of development in SCNT embryos (Supplemental Figure S2).

## Discussion

Oxidative stress can induce various types of molecular and cellular damage in pre-implantation embryos, resulting in defective embryonic development and reduction in overall growth^22^. The accumulation of ROS can induce oxidative stress, leading to damage of proteins and nucleic acids^43^. Our study showed that melatonin promoted the development of porcine SCNT embryos by enhancing DNA damage repair during somatic cell reprogramming after H_2_O_2_-induced oxidative stress. This suggests that melatonin protects against oxidative stress during SCNT embryo development.

The proportion of embryos undergoing blastocyst formation considerably increased when the SCNT embryo culture medium was supplemented with melatonin at a concentration of 1 μM. Furthermore, treating porcine SCNT embryos with melatonin had a positive effect on the expression of pluripotency markers and cell proliferation as well as on inhibition of apoptosis in the blastocysts. These results support the hypothesis that melatonin improves the developmental competence of SCNT embryos, which is consistent with results of previous reports^15, 37, 44^. Oxidative stress has highly deleterious effects on pre-implantation embryos^22^. Oxidative stress is characterized by an overproduction of free radicals, which can disrupt the balance of ROS and antioxidants under normal physiological conditions^45^. The porcine embryos are highly sensitive to damage caused by ROS, including that elicited by exposure to H_2_O_2_^46^. Thus, reducing excessive ROS production using antioxidants may effectively improve porcine SCNT embryo development. Melatonin, a pineal indoleamine, has been identified as a potent inhibitor of ROS production, which may account for its antioxidant activities^47^. Previous researches showed that supplementation of melatonin in culture media effectively prevented H_2_O_2_-induced ROS accumulation and inhibited apoptotic signals in rat brain astrocytes^37, 48^. Consistent with this, our data also showed that melatonin not only prevented the increase in intracellular ROS induced by H_2_O_2_ exposure but also considerably increased the levels of GSH in porcine SCNT embryos. Besides quenching ROS directly, maintaining stable mitochondrial function also contributes to the powerful antioxidant properties of melatonin^49^. Mitochondria play a pivotal role during early embryonic development, and mitochondrial dysfunction is associated with failure of the development of oocytes and embryos^41^. ΔΨm is commonly used as an indicator of mitochondrial function and cellular viability in embryos^50^. It reflects the activity of the hydrogen ion pump within the membrane-bound electron transport chain as well as the oxidative phosphorylation, which is the driving force behind adenosine triphosphate (ATP) production. In our study, administration of H_2_O_2_ led to a dissipation of ΔΨm, and this effect was blocked by pre-incubation with melatonin. These results are consistent with the findings of a previous study^51^. This may account for the high developmental competence observed for SCNT embryos after melatonin treatment.

Many embryos enter into a transient cell cycle arrest when they experience oxidative stress^52^. This process is activated by DNA damage before the induction of apoptosis^53^. H_2_O_2_-induced DNA damage has been observed in many types of cells undergoing apoptosis, and it is considered to be a biochemical hallmark of apoptosis^22, 54^. Melatonin is a highly lipophilic substance that easily penetrates organic membranes^47^. Therefore, it protects important intracellular structures such as mitochondria and DNA from oxidative damage^39^. Exogenous melatonin supplementation has been shown to effectively prevent oxidative stress-induced DNA damage in human spermatozoa^55^ and bovine oocytes^39^. Therefore, melatonin may enhance the developmental competence of porcine SCNT embryos by reducing the extent of DNA damage. To test this hypothesis, we evaluated the effectiveness of melatonin in reducing the amount of DNA damage in porcine SCNT embryos. The protective effect of melatonin after H_2_O_2_ exposure was investigated by nuclear staining of γH2A.X, which is a widely used marker of DNA damage in cells^25^. Specifically, γH2A.X acts as a regulator of cell cycle progression by inhibiting DNA replication in embryos^22^ and stem cells^56^. Our previous study showed that DNA damage decreased embryo development before the blastocyst stage^57^. In this study, we showed that melatonin treatment consistently reduced the expression of γH2A.X in porcine SCNT embryos at 3 and 5 days of development after H_2_O_2_ treatment. The protective effect of melatonin against DNA damage was also confirmed by the comet assay. It is likely that the mitigation of DNA damage is related to the ability of melatonin to increase catalase activity. These findings suggest that melatonin treatment has a significant effect on H_2_O_2_-induced DNA damage, protecting embryonic cells from nuclear fragmentation during oxidative stress. Histone modifications associated with somatic cell reprogramming such as deacetylation of histones H3 and H4 were observed during SCNT embryo development^8^. Global H3K9ac was rapidly and reversibly reduced in response to DNA damage^58^. A previous study showed that global H3K9ac and H3K8ac levels decreased after H_2_O_2_-induced oxidative stress^59^. In addition, global histone acetylation is also reduced under short-term oxidative stress, which could be mediated by an upregulation in the activity of class I/II HDAC^60^. A previous study suggested that the treatment with scriptaid improved the development of SCNT embryos by enhancing DNA damage repair^30^. The pig zygotic gene activation (ZGA) was confirmed to occur at the four-cell stage via genome-wide gene expression analysis, compensating for the loss of maternal transcripts^61^. Therefore, we quantified the levels of global H3K9ac in porcine SCNT embryos at the one- and two-cell stages. In this study, global H3K9ac levels were higher in melatonin-treated SCNT embryos than those in non-treated embryos. Therefore, it is likely that melatonin treatment facilitates DNA damage repair by increasing histone acetylation under conditions of oxidative stress.

In mammals, two molecular pathways are involved in DNA damage repair: HR and NHEJ^62, 63^. In response to DNA damage, activated ataxia telangiectasia mutated (ATM) and ataxia telangiectasia Rad3-related (ATR) could phosphorylate histone H2A.X at the sites of DNA DSB formation^64^. The phosphorylation of histone H2A.X is important for DSB repair, because H2A.X anchors critical initiator proteins that are required for both the HR and NHEJ pathways, which can be co-localized with γH2AX at the DSB sites^65, 66^. In order to further investigate the effect of melatonin treatment on DSB repair in porcine SCNT embryos, we assessed the expression of several genes involved in the HR and the NHEJ pathways in porcine SCNT embryos at 5 and 7 days of development. We observed that melatonin treatment caused an overall decrease in the expression of genes involved in the HR and NHEJ repair pathways at 5 days of development in SCNT embryos after H_2_O_2_ exposure. The loss of expression of DNA repair genes indicated that SCNT embryos treated with melatonin had less DNA damage at 5 days of development. However, no expression differences were observed for these genes in SCNT embryos at 7 days of development. Therefore, our results reveal a protective role of melatonin during the *in vitro* development of porcine SCNT embryos, suggesting that it inhibits DNA damage caused by oxidative stress.

In conclusion, this study shows that melatonin enhances the developmental competence of porcine SCNT embryos by preventing oxidative stress-induced DNA damage. Melatonin directly inhibits DNA damage induced by oxidative stress in the *in vitro* culture of these embryos. Our results improve the understanding of the mechanisms by which melatonin affects the *in vitro* developmental potential of embryos. We provided evidence to support this hypothesis by demonstrating that oxidative stress induced DNA damage, and melatonin mitigated these effects. On the basis of these findings, we propose that chromatin remodeling during cell reprogramming after SCNT is not only important for resetting the epigenetic program, but it also promotes DNA damage repair and preserves the genomic integrity vital for normal embryonic development.

## Materials and Methods

This research was carried out in accordance with the Ethics Committee of Chungbuk National University. All chemicals used in this study were purchased from Sigma Chemical Company (St. Louis, MO, USA) unless otherwise indicated.

### Collection of porcine oocytes and *in vitro* maturation

Porcine ovaries were obtained from slaughtered pigs at a local slaughterhouse and transported to the laboratory in sterile saline (0.9% NaCl) containing 75 μg/mL penicillin G and 50 μg/mL streptomycin sulfate at 35–37 °C within 2 h in a thermos flask. Follicles 3–6 mm in diameter were aspirated. Cumulus-oocyte complexes (COCs) that were surrounded by a minimum of three cumulus cells were selected for culture. The COCs were washed three times in Tyrode’s Lactate 4-(2-hydroxyethyl)-1-piperazineethanesulfonic acid (TL-HEPES) supplemented with 0.1% polyvinyl alcohol (PVA, w/v) and 0.05 mg/mL gentamycin. COCs were matured in tissue culture medium 199 (TCM-199; Invitrogen, Carlsbad, CA, USA) supplemented with 10% (v/v) porcine follicular fluid, 1 μg/mL insulin, 75 μg/mL kanamycin, 0.91 mM Na pyruvate, 0.57 mM L-cysteine, 10 ng/mL epidermal growth factor, 0.5 μg/mL follicle stimulating hormone, and 0.5 μg/mL luteinizing hormone for 40–42 h at 38.5 °C in a humidified atmosphere of 5% CO_2_.

### SCNT procedure and embryo culture

Porcine fetal fibroblasts were used as nuclear donors and cultured as previously described^67^. For enucleation, denuded oocytes were enucleated by aspirating the polar body and metaphase chromosomes in a small amount (<15% of the oocyte volume) of cytoplasm using a 25-µm beveled glass pipette (Humagen, Charlottesville, VA, USA). After enucleation using a fine injecting pipette, a single donor cell was inserted into the perivitelline space of the enucleated oocyte. Membrane fusion was induced by applying an alternating current field of 2 V cycling at 1 MHz for 2 s, followed by a DC pulse of 200 V/mm for 20 μs, using a cell fusion generator (LF201; Nepa Gene, Chiba, Japan). Following fusion, the reconstructed embryos were placed in bicarbonate-buffered porcine zygote medium 5 (PZM-5) containing 0.4 mg/mL bovine serum albumin (BSA) for 1 h prior to activation. Activation was performed by applying DC pulses of 150 V/mm for 100 µs in 297 mM mannitol containing 0.1 mM CaCl_2_, 0.05 mM MgSO_4_, 0.01% PVA (w/v), and 0.5 mM HEPES. After activation, the reconstructed embryos were cultured in bicarbonate-buffered PZM-5 containing 0.4 mg/mL BSA and 7.5 μg/mL CB for 3 h to suppress extrusion of the pseudo-second polar body. After culture, the reconstructed embryos were thoroughly washed and cultured in bicarbonate-buffered PZM-5 supplemented with 0.4 mg/mL BSA in 4-well dishes for 7 days at 38.5 °C under 5% CO_2_ without changing the medium. The development of the reconstructed embryos into blastocysts was examined 7 days after activation.

### Immunofluorescence staining

Immunostaining was performed according to our previously described^9^. An antibody used to detect γH2A.X (1:100; #2577) was purchased from Cell Signaling Technology (Beverly, MA). Antibodies used to detect octamer-binding transcription factor 4 (OCT4) (1:100; sc-8628) and SOX2 (1:100; sc-17320) were purchased from Santa Cruz Biotechnology (Santa Cruz, CA). Anti-H3K9ac antibody (1:500; ab10812) was purchased from Abcam (Cambridge, MA). The embryos were examined under a confocal laser scanning microscope (Zeiss LSM 510 and 710 META; Zeiss, Oberkochen, Germany). Fluorescence intensities were quantified using ImageJ software (National Institutes of Health, Bethesda, MD, USA)^68^. The number of γH2A.X foci was quantified using the Zeiss software, and foci larger than 0.3 μm^3^ were deemed to be sites of DSB^25, 65^.

### 5-Bromo-2′-deoxyuridine (BrdU) analysis

Cell proliferation was assessed by performing a BrdU assay^69^. Blastocysts were incubated with 100 μM BrdU in a humidified atmosphere of 5% CO_2_ at 38.5 °C for 6 h. These blastocysts were washed three times with Dulbecco’s phosphate-buffered saline (PBS) containing 0.05% Tween 20 (PBS-T), fixed in ice-cold methanol for 20 min, and permeabilized with 0.2% Triton X-100 for 2 min. The blastocysts were then washed with PBS-T and treated with 2 N HCl for 30 min. Next, the blastocysts were washed and incubated with a mouse anti-BrdU monoclonal antibody (Sigma; B2531) diluted 1:10 at 4 °C overnight. After washing with 0.1% BSA prepared in PBS, the blastocysts were incubated with a rabbit anti-mouse IgG Alexa Fluor 568-conjugated polyclonal antibody (1:200; Cat: A-11061; Invitrogen) at room temperature for 1 h. After extensive washing with PBS-T, embryos were counterstained with 10 μg/mL Hoechst 33342 for 15 min, mounted on glass slides, and examined under a confocal laser scanning microscope. Proliferating cells were counted after merging multilayer cut planes using ImageJ software.

### Terminal deoxynucleotidyl transferase (TdT) 2′-deoxyuridine, 5′-triphosphate (dUTP) nick end labeling (TUNEL) assay

For the TUNEL assay, blastocysts were fixed in 3.7% paraformaldehyde for 1 h. After fixation, the blastocysts were permeabilized by treatment with 0.1% Triton X-100 for 1 h at 37 °C. The blastocysts were washed twice in PBS-PVA and incubated in the dark for 1 h at 37 °C with TdT and fluorescein-conjugated dUTPs (*In Situ* Cell Death Detection kit; Roche, Mannheim, Germany). The blastocysts were stained with 10 μg/mL Hoechst 33342 for 15 min, mounted onto glass slides, and examined under the confocal laser scanning microscope. The cells and apoptotic nuclei were quantified after creating z-stack projections. The percentage of apoptotic nuclei was calculated as the number of apoptotic nuclei/total number of nuclei ×100.

### Comet assay

Comet assays were performed using the OxiSelect Comet Assay kit (Cat: STA-350; Cell Biolabs, USA), according to the manufacturer’s instructions, with minor changes. Briefly, the OxiSelect comet agarose bottle was heated to 90–95 °C in a water bath for 20 min. The agarose bottle was transferred to a 37 °C water bath for 20 min. The zona pellucida was removed using 1 mg/mL pronase, and the embryos were transferred to ice-cold PBS. Samples were combined with pre-warmed comet agarose at a 1:10 ratio (v/v), and 75 μL/well was transferred onto an OxiSelect comet slide. The samples were lysed in pre-chilled lysis buffer for 4 h at 4 °C in the dark. After lysis, the slides were carefully transferred to a pre-chilled alkaline solution for 30 min at 4 °C in the dark. Subsequently, the slides were carefully transferred to a horizontal electrophoresis chamber filled with cold Tris/borate/ethylenediaminetetraacetic acid (TBE) buffer and electrophoresed for 20 min at 25 V. Slides were washed with pre-chilled water for 5 min and cold 70% ethanol for 5 min. Slides were stained with Vista Green DNA Dye for 15 min and examined using a fluorescent microscope (Nikon Eclipse TE200; Nikon Corp., Tokyo, Japan) with a FITC filter. The comet tail lengths were measured in individual embryos using CASP (ver. 1.2.3beta2; Zbigniew Koza, Poland).

### Intracellular ROS and GSH levels

To determine intracellular ROS levels, embryos were incubated for 15 min in PBS-PVA medium containing 10 µM 2′,7′-dichlorodihydrofluorescein diacetate. To determine intracellular GSH levels, embryos were incubated for 30 min in PBS-PVA medium containing 10 μM 4-chloromethyl-6,8-difluoro-7-hydroxycoumarin (CMF2HC) (Invitrogen). Fluorescent signals were captured as a tagged image file format (TIFF) using a digital camera (DP72; Olympus, Tokyo, Japan) connected to the fluorescence microscope (IX70, Olympus, Tokyo, Japan). The same procedures, including incubation, rinsing, mounting, and imaging, were followed for all groups of embryos. ImageJ software was used to analyze the fluorescent intensities of the embryos.

### ΔΨm assay

The ΔΨm assay was performed using the ΔΨm-sensitive fluorescent probe 5,5′,6,6′-tetrachloro-1,1′,3,3′-tetraethyl-imidacarbocyanine iodide (JC-1; Thermo Fisher Scientific, San Jose, CA, USA). Briefly, embryos were incubated in PBS-PVA containing 2 μM JC-1 for 30 min. The ΔΨm was calculated as a ratio of red florescence (corresponding to activated mitochondria; J-aggregates) to green fluorescence (corresponding to less active mitochondria; J-monomers)^70^. Fluorescence was visualized using the digital camera connected to the fluorescence microscope. The resulting images were processed using the ImageJ software. The fluorescence intensity per pixel was automatically computed by ImageJ.

### Reverse transcription quantitative polymerase chain reaction (RT-qPCR) analysis

Total RNA was extracted from 15 embryos, using the Dynabeads mRNA DIRECT kit (Invitrogen), according to the manufacturer’s instructions. First-strand cDNA was synthesized by reverse transcription of mRNA using the Oligo(dT) 12-18 primer and SuperScript TM III reverse transcriptase (Invitrogen). RT-qPCR was performed using SYBR Green, a fluorophore that binds double-stranded DNA, in a final reaction volume of 20 µL using the CFX96 touch RT-PCR detection system (Bio-Rad, Hercules, CA, USA). Gene expression was quantified by the 2^−ΔΔCt^ method, with normalization to the expression levels of glyceraldehyde-3-phosphate dehydrogenase (*GAPDH*). The PCR primers used to amplify each gene are listed in Supplemental Table S1.

## Electronic supplementary material

Supplementary Information
