# Supplementary material for: Melatonin enhances the developmental competence of porcine somatic cell nuclear transfer embryos by preventing DNA damage induced by oxidative stress
Source: Sci Rep. 2017 Sep 11;7:11114. doi: 10.1038/s41598-017-11161-9 (PMC5593819; doi:10.1038/s41598-017-11161-9)
Supplement: Supplementary file 1 — Supplementary Information [file 41598_2017_11161_MOESM1_ESM.pdf]

Melatonin enhances the developmental competence of porcine somatic cell nuclear transfer embryos by preventing DNA damage induced by oxidative stress

Shuang Liang[1, 2], Yong-Xun Jin[1], Bao Yuan[1], Jia-Bao Zhang[1*] & Nam-Hyung Kim[1, 2*]

[1] Department of Laboratory Animal Center, College of Animal Sciences, Jilin University, Changchun, 130062, China.
[2] Department of Animal Science, Chungbuk National University, Cheongju, 361-763, Republic of Korea.

[*]Corresponding authors. Nam-Hyung Kim (email: nhkim@chungbuk.ac.kr) and Jia-Bao Zhang (email: zjb515@163.com)

Supplemental Figure Legends

Supplemental Figure S1. To determine the oxidative damage, the reconstructed embryos were exposed to different concentrations of H_2O_2 (0–200 μM) for 30 min, washed extensively, and cultured in bicarbonate-buffered porcine zygote medium 5 (PZM-5) supplemented with 0.4 mg/mL bovine serum albumin (BSA) until day 7. To determine the protective effect of melatonin against oxidative stress, the reconstructed embryos were pre-treated for 3 h with 1 μM melatonin followed by addition of 100 μM H_2O_2 for 30 min and subsequently cultured in presence of melatonin until day 7. H_2O_2 treatment reduced the developmental potential of embryos in a dose-dependent manner. Melatonin treatment rescued the developmental potential of embryos after H_2O_2 exposure. The numbers of embryos examined in each group are shown in the bars. Data are expressed as the mean ± standard deviation (SD) from at least three separate experiments. * $p < 0.05$.

Supplemental Figure S2. Relative mRNA levels of damage-related genes of the homologous recombination (HR) and non-homologous end-joining (NHEJ) pathways

in somatic cell nuclear transfer (SCNT) embryos at day 7 of development. Data are expressed as the mean ± standard deviation (SD) from at least three separate experiments.

Supplemental Figure S1.

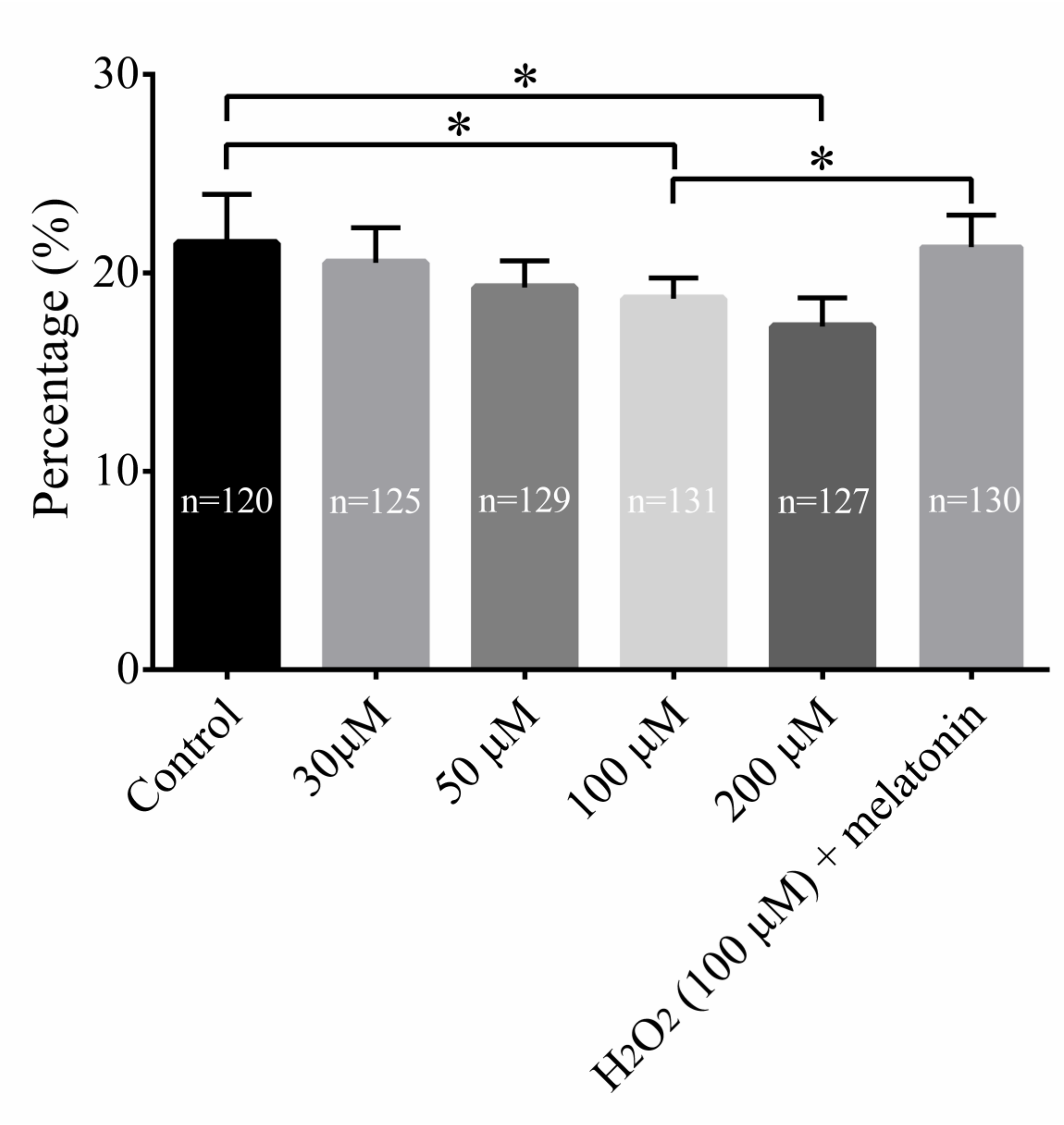

Supplemental Figure S2.

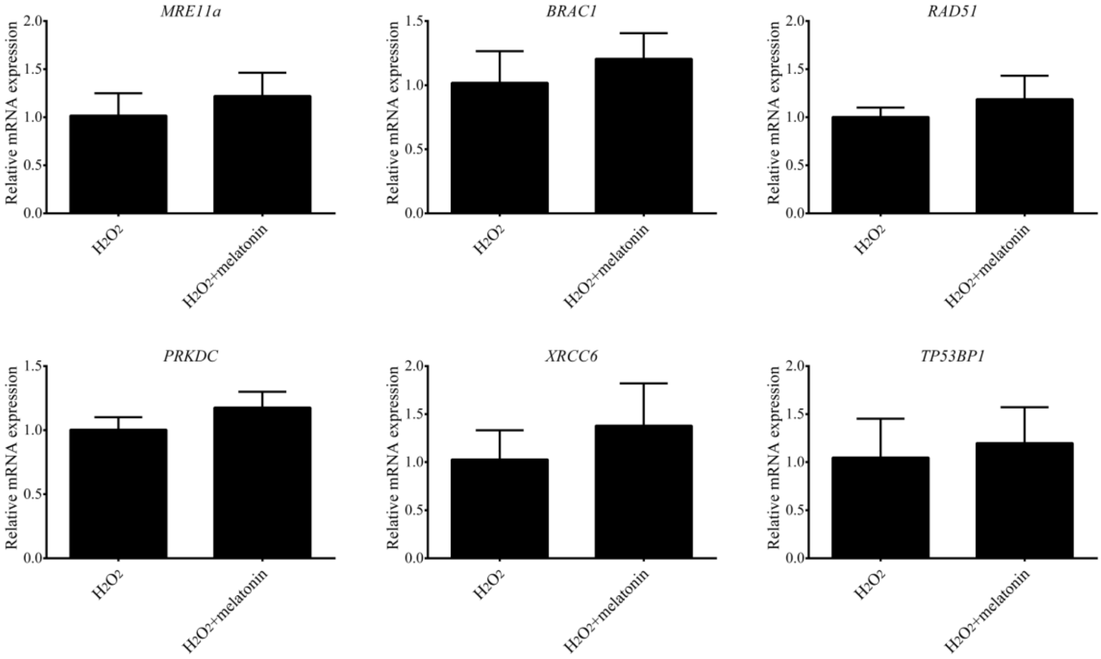

Supplemental Table S1: Primers used in this study.

Gene	Primer sequences (5'-3')	Genebank accession no.
MRE11A	F: GGAGGATGTTGTCCTGGCTG<br>R: AGACGTTCCCGTTCTGCATT	XM_003129789.2
BRCA1	F: TGCTAAATCCGGAACAAAACACA<br>R: CTGGTGGAACGATCCAGAGAT	XM_003358030.1
RAD51	F: CTTCGGTGGAAGAGGAGAGC<br>R: CGGTGTGGAATCCAGCTTCT	NM_001123181.1
PRKDC	F: ATTCTTTGTCGGGAGCAGCA<br>R: CCTAGCTGTGTGGCACATGA	XM_001925309.4
XRCC6	F: ACGGAAGGTGCCCTTTACTG<br>R: TGCAGCACTGGGTTCTCAAA	NM_001190185.1
TP53BP1	F: GGGAAAGGGGGAGTTCGTG<br>R: CTCACGCTCGTGCTAGAGAT	XM_001925938.4
GAPDH	F: TTCCACGGCACAGTCAAG<br>R: ATACTCAGCACCAGCATCG	NM_001206359

F: Forward; R: Reverse.
